# Case Report: A case report of dry tap during ventriculostomy

**DOI:** 10.12688/f1000research.6750.2

**Published:** 2015-10-02

**Authors:** Sunil Munakomi, Binod Bhattarai

**Affiliations:** 1 Department of Neurosurgery, College of Medical Sciences, Bharatpur, 44207, Nepal

**Keywords:** Dry tap, Pneumocephalus, Management

## Abstract

Pneumocephalus following ventriculoperitoneal (VP) shunt insertion is an exceptionally rare occurrence. We report such an event after attempting ventricular puncture (ventriculostomy) for VP shunt insertion and then discuss the management of the same. Dry tap can lead to multiple attempts for ventriculostomy with the associated added risks of complications, as well as complicating the subsequent management. In addition, there is an increased risk of tension pneumocephalus, seizure and shunt failure due to a blockage by air bubbles. Our patient presented with features of raised intracranial pressure two months following craniotomy and evacuation of traumatic subdural hematoma. External ventricular puncture revealed egress of CSF under pressure. Upon attempting VP shunting for post-traumatic hydrocephalus, we experienced dry tap during ventricular puncture that complicated further management. We placed the proximal shunt in the presumed location of the foramen of Monro of ipsilateral frontal horn of lateral ventricle and did not remove the external ventricular drain. Post-operative CT scan revealed pneumoventriculi as the cause for the dry tap during ventricular puncture. Patient was managed with 100% oxygen. He showed gradual improvement and was later discharged. This case shows that variations in the procedure, including head down positioning, adequate cruciate dural incision prior to cortex puncture, and avoiding excessive egress of CSF can help to prevent such complications.

## Introduction

Pneumocephalus is defined as the presence of air within the calvarium. It often follows trauma but is also a common sequelae of intracranial surgery ^1, 2^. Tension pneumocephalus is a life-threatening emergency that necessitates immediate surgical intervention ^3^. It is rarely reported after cerebrospinal fluid (CSF) diversion procedures ^4, 5^. We present a rare case of tension pneumocephalus, resulting in dry tap during ventriculostomy and discuss its subsequent management.

## Case report

Herein we report a case of a 35-year-old male from Nawalparasi, Nepal, who had undergone a craniotomy and evacuation of acute subdural hematoma following an automobile accident 2 months before admission to our institution. He presented with complaints of an abnormal gait, with a tendency to fall backwards and also with features of frontal lobe-related incontinence. There were no significant past medical illnesses. He was taking Sodium Valproate (300 mg oral three times daily) as seizure prophylaxis following the traumatic head injury and surgical intervention for the same 2 months previously. Fundus examination revealed the presence of papilledema. A head computerized tomography (CT) scan revealed the presence of evolving hydrocephalus. To rule out hydrocephalus *ex vacuo* due to volume loss and changes in CSF dynamics subsequent to the previous accident, external ventricular drainage (EVD) was placed which revealed egress of CSF under pressure. The reasons for opting to choose EVD prior to VP shunting are threefold. Firstly, we had to rule out post traumatic hydrocephalus *ex vacuo* by measuring the opening pressure of the CSF egress and looking for the neurological improvement in the patient following CSF diversion. Secondly, since we not have the programmable VP shunting available, we need to appropriately choose the Chabra shunt depending on the opening pressure so as to prevent either over drainage or under drainage of CSF. Lastly, since it was a post traumatic case, we need to measure the CSF protein (as it may be increased from the lysed traumatic subarachnoid blood) and also we need to rule out subclinical meningitis. Both these may be the reasons for shunt failure. Following EVD, the patient showed gross improvement in his previous deficits. CSF sugar and protein was within range. Gram stain was negative for any bacteria. Repeat CT scan post EVD did not reveal any pneumocephalus. Thereafter he was scheduled for insertion of a VP shunt. EVD was clamped for 6 hours prior to the procedure to facilitate the ventricular tap. During insertion of the VP shunt, there was dry tap during an attempt of ventriculostomy from the Kocher’s point. We made two further attempts to ensure the correct trajectory of the shunt end and also to reflush the shunt end to prevent blockage due to blood clots and cell debris. We placed the shunt tip in the presumed location of the foramen of Monro of the frontal horn of ipsilateral lateral ventricle. We did not remove the EVD, hoping that it would act as a safety channel for CSF bypass had we missed the correct trajectory for the VP shunt.

**Figure 1.**
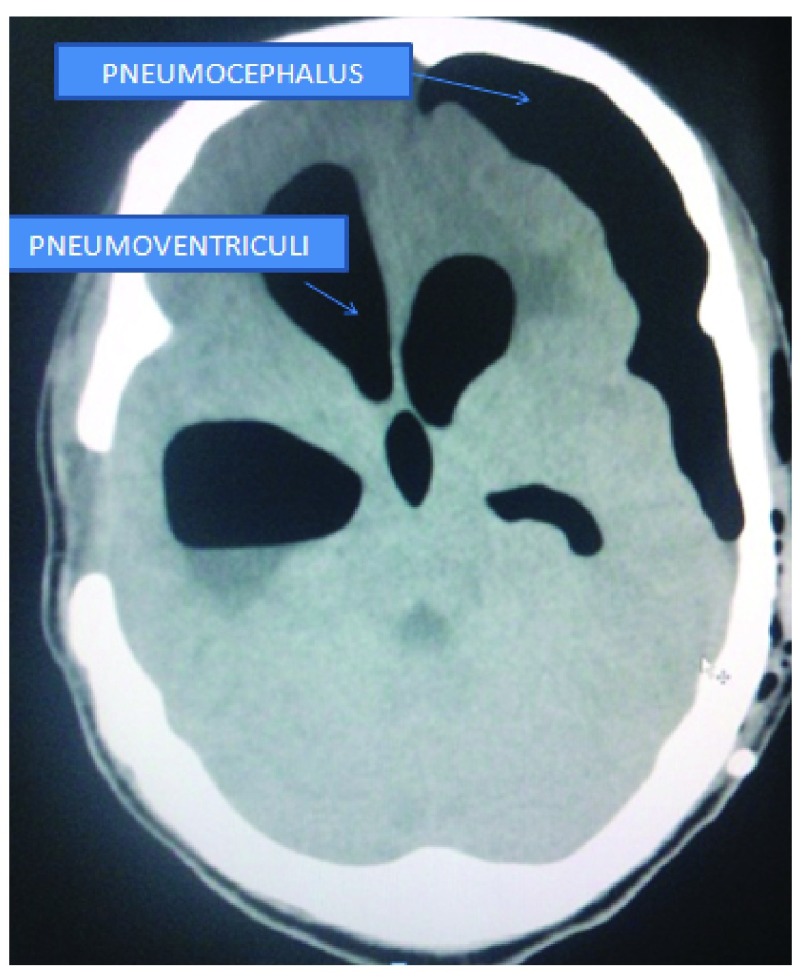
CT image showing presence of pneumocephalus and pneumoventriculi.

**Figure 2.**
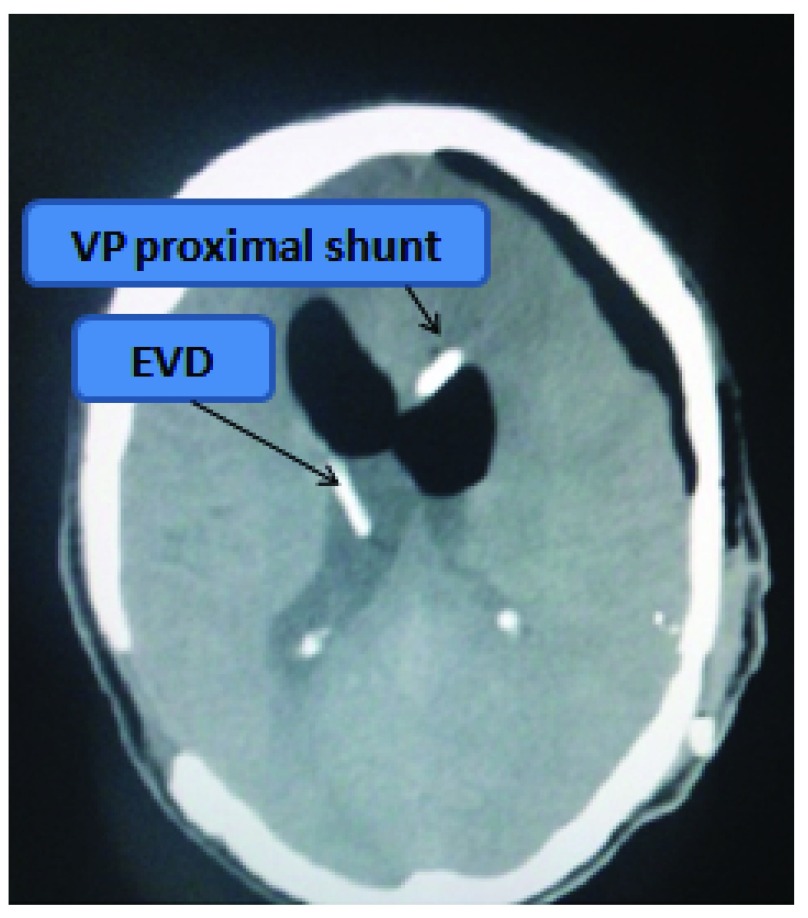
CT image showing location of EVD and VP Shunt proximal end.

**Figure 3.**
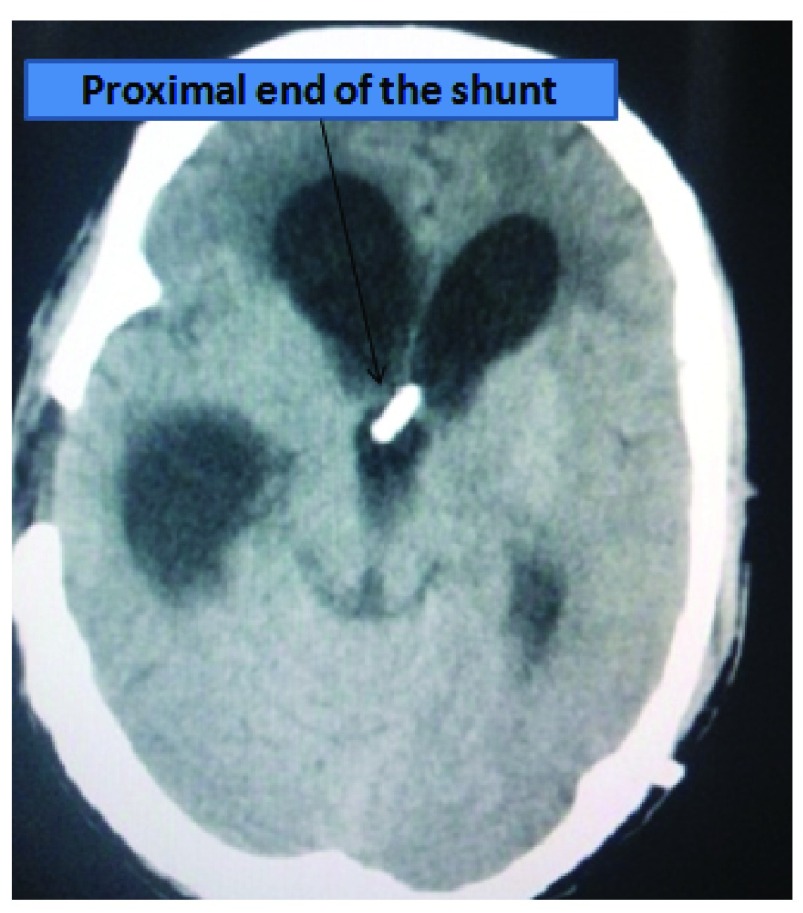
CT image confirming location of proximal end of VP shunt.

A postoperative scan revealed the presence of tension pneumocephalus and pneumoventriculi ( Figure 1 and Figure 2). The patient was managed with 100% oxygen for 3 days and was continued on antiepileptic medications at the same dose intravenously. Stringent neurological monitoring was undertaken to evaluate early neurological deterioration due to tension pneumocephalus. Pupils were routinely assessed to look for hippus (a clinical marker of epilepsy). Patient was extubated the following morning. A repeat CT scan on the 6 ^th^ day post-operation showed that the proximal shunt was in the third ventricle ( Figure 3) and there was complete resolution of the condition. The EVD was subsequently removed with no neurological deterioration of the patient on 7 ^th^ day after operation. The patient then started to walk with support from the 8 ^th^ day post-operation, and he slowly improved in gait. Patient went home walking with minimal support on the 14 ^th^ day post-operation. Patient had also regained his bladder control within that time. Patient returned, walking on his own 1 month later for his follow up in the outpatient department His gait was normal with no features of retropulsion. The shunt chamber was functioning well and his bowel habits were normal. Compliance in continuation of Sodium valproate therapy (at the aforementioned dose) was also ensured.

## Discussion

Pneumocephalus usually occurs after head trauma, skull base fractures, and associated CSF fistulas ^1^. The incidence of this entity was reported to be as high as 100% following supratentorial craniotomies ^6^. On the other hand, tension pneumocephalus is a neurosurgical emergency that requires rapid surgical intervention. Pneumocephalus as a complication of CSF diversion procedures is rare ^5^. Diagnosis is mainly based on clinical examination and computerized tomography (CT) scan ^4^. Two CT findings that characterise the condition – the ‘Mount Fuji’ sign and the ‘air bubble’ sign - have been described by Ishiwata *et al.* ^7^.

There are two factors that are thought to be responsible for tension pneumocephalus development. The first is a decrease in intracranial pressure due to a sudden egress of CSF; the second is the presence of a craniodural defect that works as a one way valve allowing air inflow to the intracranial space and preventing outflow ^4^. It is claimed that moderate cerebral atrophy might play a role ^1^.

The duration of the shunt surgery must be as short as possible and CSF leakage during the connection of the shunt system must be avoided. Another factor that can lead to undesirable outcomes can be introduced during the puncturing of the cortex. Adequate cruciate incision must be given to prevent the passage of environmental air into the subdural space. Filling the subdural space on the ventriculostomy site with irrigation fluid until overflowing might help the outflow of air from the intracranial vault, reducing the risk of this rare complication. Cortical atrophy may have also had an effect on isolated air collection within the subdural space. In our case, another remote possibility for the development of pneumocephalus would be any leak in the closed drainage system of the previous EVD drain. Properly layered closure of the skin in VP shunt surgery is the most important factor for prevention of this rare complication.

The pneumocephalus in our case most likely would have occurred either during trephination of burr hole or while incising the dura prior to ventricular tap. Other probable reasons were ruled out since the EVD was clamped (as open EVD would have caused resulted in negative pressure and thereby pneumocephalus) and we also ensured the air locked system of the EVD circuit (as breach in the same would let the air get sucked in).

The dry tap, as seen in our case, can lead to multiple attempts to attain the correct shunt trajectory, thereby increasing the risk of false trajectories and track hematomas. If there had been no EVD, then this would have led to termination of the procedure, thereby adding to the morbidity and risk of subsequent surgery. One alternative to our approach would be the use of intra-operative CT scan to ensure the diagnosis. Unfortunately this is not currently possible in developing countries like ours. We can, if available, take help of neuro-navigation tools to ensure the correct trajectory to the ventricles even in cases were in pneumocephalus occurs. This can reduce the added burden of subsequent surgeries and associated risk of anesthesia.

There is also risk of seizure and rapid neurological deterioration due to tension pneumocephalus. Once this occurs, close monitoring of the patient, rapid and accurate identification of tension pneumocephalus, and immediate surgical intervention is life-saving. Gore *et al.* ^8^ have advocated the use of 100% oxygen for rapid resolution of pneumocephalus.

In conclusion, though VP shunting is one of the most common surgical procedures performed in neurosurgery, strict adherence to basic principles should be followed during the procedure so as to prevent avoidable complications such as in our case which may in times lead to sudden deterioration in the patient and also add to diagnostic and therapeutic dilemma to the concerned surgeons. One advantage in this case was the presence of an EVD as a safety bypass for CSF diversion. The limitations of our approach can be attributed to the unavailability of intra-operative CT scan and neuronavigation techniques which would have aided in early diagnosis and management in this scenario.

## Consent

Both written and verbal informed consent for publication of images and clinical data related to this case was sought and obtained from the wife of the patient.

## References

[1] Barada W Najjar M Beydoun A: Early onset tension pneumocephalus following ventriculoperitoneal shunt insertion for normal pressure hydrocephalus: a case report. *Clin Neurol Neurosurg.* 2009;111(3):300–302. 10.1016/j.clineuro.2008.10.013 19185417

[2] Kawajiri K Matsuoka Y Hayazaki K: Brain tumors complicated by pneumocephalus following cerebrospinal fluid shunting--two case reports. *Neurol Med Chir (Tokyo).* 1994;34(1):10–14. 10.2176/nmc.34.10 7514746

[3] Monas J Peak DA: Spontaneous tension pneumocephalus resulting from a scalp fistula in a patient with a remotely placed ventriculoperitoneal shunt. *Ann Emerg Med.* 2010;56(4):378–381. 10.1016/j.annemergmed.2010.05.030 20619934

[4] Tuğcu B Tanriverdi O Günaldi O: Delayed intraventricular tension pneumocephalus due to scalp-ventricle fistula: a very rare complication of shunt surgery. *Turk Neurosurg.* 2009;19(3):276–280. 19621294

[5] Ugarizza LF Cabezudo JM Lorenzana LM: Delayed pneumocephalus in shunted patients. Report of three cases and review of the literature. *Br J Neurosurg.* 2001;15(2):161–167. 10.1080/02688690151127482 11360384

[6] Reasoner DK Todd MM Scamman FL: The incidence of pneumocephalus after supratentorial craniotomy. Observations on the disappearance of intracranial air. *Anesthesiology.* 1994;80(5):1008–1012. 10.1097/00000542-199405000-00009 8017640

[7] Ishiwata Y Fujitsu K Sekino T: Subdural tension pneumocephalus following surgery for chronic subdural hematoma. *J Neurosurg.* 1988;68(1):58–61. 10.3171/jns.1988.68.1.0058 3335913

[8] Gore PA Maan H Chang S: Normobaric oxygen therapy strategies in the treatment of postcraniotomy pneumocephalus. *J Neurosurg.* 2008;108(5):926–929. 10.3171/JNS/2008/108/5/0926 18447708

