# Supplementary material for: Case Report: A case report of dry tap during ventriculostomy
Source: F1000Res. 2015 Oct 2;4:188. Originally published 2015 Jul 7. [Version 2] doi: 10.12688/f1000research.6750.2 (PMC4871012; doi:10.12688/f1000research.6750.2)
Supplement: Peer review history

# Peer review: Referee response for version 2

**DOI:** 10.5256/f1000research.7698.r13750

**Published:** 2016-05-13

**Authors:** Guru Dutta Satyarthee, Luis Rafael Moscote-Salazar

**Affiliations:** 1 Department of Neurosurgery, All India Institute of Medical Sciences, New Delhi, Delhi, India; 2 Neurosurgery-Critical Care, RED LATINO. Latin American Trauma & Intensive Neuro-Care Organization, Bogota, Colombia

The authors provide important guidelines for the evaluation and management of hydrocephalus developing in the post-craniotomy follow-up period after surgical traumatic acute subdural evacuation. The authors presented an interesting case, which presented with gait abnormality associated with falling back tendency and underwent craniotomy surgery two months earlier for evacuation acute subdural hematoma. A cranial computed tomography revealed presence of hydrocephalus. In the standard management of post traumatic craniotomy development of hydrocephalus, it is essential to evaluate the possible causes, degree of intracranial pressure rise, evaluating beneficial effect of cerebrospinal fluid diversion procedure i.e. ventriculoperitoneal shunt or thecoperitoneal shunt, assessment of CSF protein level, and exclusion of associated indolent meningitis as possible causes.

Ideally for excluding ex-vacuo post-traumatic hydrocephalus development, CSF sampling can be done through lumbar drainage or by placing external ventricular drainage and assessing initial CSF egress pressure with manometry for selection of pressure range and most suitable type of future shunt requirement, cytological, protein level for suitability of shunt functioning after shunt insertion; as very high protein level can be relative contraindication for shunt, biochemical study and culture sensitivity studies to exclude infective pathology and assessing Mini mental scale for cognitive assessment and gait changes . The patient shows improvement following CSF drainage CSF diversion surgery in the form of thecoperitoneal or ventriculoperitoneal shunt, however the former is the preferred choice. ^1^

 Development of pneumocephalus requires communication between arachnoid membrane and associated defect in the dura, calvarium and communication to the external environment. As CSF egresses, air enters the intracranial cavity and development of pneumocephalus. The “ball valve” mechanism was postulated by Dandy, as air travels in only one direction ^2^. After air entering the cranial cavity leads to rise in the intracranial pressure ^3^.  

The other postulates for tension pneumocephalus is the “Inverted-soda-bottle effect”, proposed by Horowitz ^4^. The author postulated negative intracranial pressure resulting from excessive loss of CSF through an iatrogenic lumbar drain or settling into the distendable spinal subarachnoid space or simply drainage via normal pathways with physiologic activity such as inspiration or the Valsalva manoeuvre ^4^. However, when there are fistulous communications between the intracranial and outer environment, air can enter the intracranial space as a consequence of negative pressure gradient ^3^^,^^5^^-^^6^. As the authors' case had EVD, that might have unfortunately disconnected and leak of CSF led to development of tension pneumocephalus. In cases of dry tap, insertion of the ventricular end is specially challenging and options include abandoning the procedure,  getting an intraoperative cranial CT scan or help of an endoscope, or image guidance or infusing fluid through the EVD into the ventricle thereby distending the ventricle prior to reattempting Ventricular catheter insertion. However, dry tap is really a neurosurgical emergency needing tactful management, which depends on available facility at surgical centre including image guidance, intraoperative CT scan facility, endoscope facility and expertise of surgical team.

We have read this submission. We believe that we have an appropriate level of expertise to confirm that it is of an acceptable scientific standard.

## References

[1] AgrawalDPandeyNSinhaSGuptaDSatyartheeGSinghP: Subdural effusion with ventriculomegaly after decompressive craniectomy for traumatic brain injury: A challenging entity. *The Indian Journal of Neurotrauma*.2014;11(2) : 10.1016/j.ijnt.2014.11.001 97-102 10.1016/j.ijnt.2014.11.001

[2] DANDYW: PNEUMOCEPHALUS (INTRACRANIAL PENUMATOCELE OR AEROCELE). *Archives of Surgery*.1926;12(5) : 10.1001/archsurg.1926.01130050003001 10.1001/archsurg.1926.01130050003001

[3] LeeSHKohJSBangJSKimMC: Extensive tension pneumocephalus caused by spinal tapping in a patient with Basal skull fracture and pneumothorax. *J Korean Neurosurg Soc*.2009;45(5) : 10.3340/jkns.2009.45.5.318 318-21 10.3340/jkns.2009.45.5.318 19516955PMC2693797

[4] HOROWITZM: INTRACRANIAL PNEUMOCOELE. AN UNUSUAL COMPLICATION FOLLOWING MASTOID SURGERY. *J Laryngol Otol*.1964;78:128-34 1412627610.1017/s0022215100061910

[5] RathoreASSatyartheeGDMahapatraAK: Post-Traumatic Tension Pneumocephalus: Series of Four Patients and Review of the Literature. *Turk Neurosurg*.2016;26(2) : 10.5137/1019-5149.JTN.4238-11.1 302-5 10.5137/1019-5149.JTN.4238-11.1 26956830

[6] SatyartheeGDMahapatraAK: Tension pneumocephalus following transsphenoid surgery for pituitary adenoma - report of two cases. *J Clin Neurosci*.2003;10(4) :495-7 1285289510.1016/s0967-5868(03)00087-0

# Peer review: Referee response for version 1

**DOI:** 10.5256/f1000research.7251.r10474

**Published:** 2015-09-24

**Authors:** Roman Bosnjak

**Affiliations:** 1 Department of Neurosurgery, University Medical Centre Ljubljana, Ljubljana, Slovenia

The authors analysed dry tap during ventriculostomy in the presence of EVD inserted few days before.

Their explanations are reasonable. But there might be additional explanations.

The EVD was inserted to rule out ex vacuo hydrocephalus.  CSF egressed under pressure. Here the authors could insert VP shunt on first occasion (without previous EDV).   

There is no report on patient's condition between EVD and VP insertion.  If it improved and remained stable, no significant tension hydrocephalus is expected to evolve. It is my theory that the air was sucked into the ventricles and trapped subdurally at the time of VP shunt insertion when EDV was probably not stopped enough long time before VP insertion.   

EDV produced some minor negative pressure.  During attempts of inserting ventricular catheter of VP shunt produced sucking of the air into the ipsilateral ventricle and trapping it there.  Similarly, during trephination of a burr hole for insertion of VP shunt  and cutting the dura, some air was sucked subdurally on that side.   

As slowly evolving postraumatic  hydrocephalus is mainly of malabsorptive communicative type, lumbar drainage is less invasive attempt to reveal ex vacuo hydrocephalus than EDV. EDV or LD is stopped cca. 4-6h before inserting VP catheter to reexpand the ventricles and ease targeting.

I have read this submission. I believe that I have an appropriate level of expertise to confirm that it is of an acceptable scientific standard, however I have significant reservations, as outlined above.

# Response

**Published:** 2015-09-24

**Authors:** Sunil Munakomi

Respected sir,

Thank you for your report.

We place the EVD in our ICU in suspected cases of hydrocephalus and  then plan for the shunting if there is egress of CSF under pressure and neurological improvement in the patient. This case showed neurological improvement after EVD placement. The repeat CT after the shunt did not reveal pneumocephalus. We routinely do clamp the EVD 6 hours prior to the procedure to facilitate the tap.As you have correctly mentioned ,the pneumocephalus might have resulted either during the trephination or during corticol incision prior to ventricular tap.Sorry to have missed these points in our paper.

# Peer review: Referee response for version 1

**DOI:** 10.5256/f1000research.7251.r9362

**Published:** 2015-08-11

**Authors:** Guoyi Gao

**Affiliations:** 1 Department of Neurosurgery, Shanghai Jiao Tong University, Shanghai, China

VP shunt is the most frequent surgical technique in neurosurgery to treat hydrocephalus related with trauma, SAH, tumor and other brain diseases. Dry tap, which is a rare episode during the shunt operation, may cause confused decision and leading to complications. In this report, authors described the details of the dry tap and analysis the possible reasons behind, which is helpful for neurosurgeons to prevent this rare complication in clinical settings.

I have read this submission. I believe that I have an appropriate level of expertise to confirm that it is of an acceptable scientific standard.
